# LINC00630 promotes cholangiocarcinoma cell proliferation, migration and invasion by mediating the miR-199a/FGF7 axis

**DOI:** 10.7150/jca.66850

**Published:** 2022-01-04

**Authors:** Baiyin Zhong, Caixin Song, Qingfang He, Zhixi Chen, Qicheng Liao, Qiusheng Xiong, Shijie Wang, Yuansheng Xiao, Xing Xie, Yuankang Xie, Xiaonong Wang, Jianhong Zhang

**Affiliations:** 1 Department of Hepatobiliary Surgery, the First Affiliated Hospital of Gannan Medical University, Ganzhou China, 341000; 2 Department of General Surgery, the First Affiliated Hospital of Gannan Medical University, Ganzhou China, 341000; 3 College of Pharmacy, Gannan Medical University, Ganzhou China, 341000

**Keywords:** cholangiocarcinoma, long intergenic non-protein coding RNA 630, microRNA-199a, fibroblast growth factor 7, migration, invasion

## Abstract

Cholangiocarcinoma (CCA) is a type of cancer with a relatively low morbidity, but poor prognosis. Aberrant long non-coding RNA (lncRNA) expression has been observed in the pathological development of CCA. In the present study, lncRNA long intergenic non-protein coding RNA 630 (LINC00630) was found to be significantly upregulated in CCA tissues and cultured cells. LINC00630 expression was positively associated with histological differentiation, TNM stage and lymph node invasion. Short hairpin RNA (sh)-LINC00630 transfection could effectively decrease CCA cell proliferation, migration and invasion. Further investigations found that LINC00630 could interact with microRNA (miR)-199a, which specifically targeted fibroblast growth factor 7 (FGF7) for degradation. FGF7 overexpression restored the sh-LINC00630 transfection-induced decrease in CCA cell proliferation, migration and invasion. In conclusion, LINC00630 significantly promoted CCA cell proliferation, migration and invasion by upregulating FGF7 through miR-199a sponging.

## Introduction

Cholangiocarcinoma (CCA), the second most common type of primary liver cancer, is a type of cancer that pathologically arises from the dysregulation of bile duct epithelial cells 1. CCA-related morbidity has been significantly increasing, as confirmed by a large surveillance, epidemiology and end results database review reporting a 128% net increase in the last 40 years 2. The etiology of CCA involves several factors, such as bile duct stones, cholelithiasis and primary sclerosing cholangitis. Patients with CCA often receive treatment at the advanced stage of the disease, resulting in high recurrence and mortality rates, as well as clinical challenges 3. Although the clinical management of CCA has been improved, its prognosis remains poor 3. This is because the molecular pathogenesis of CCA remains unclear and the specific diagnostic biomarkers currently in use are insufficient.

The poor prognosis and high recurrence rate of CCA may be associated with its high invasion and migration activities. Fibroblast growth factor 7 (FGF7) acts as a ligand that interacts with FGF receptor 2 (FGFR2), mediating the pathophysiological changes induced by the diseases. FGFR2 has been implicated in the progression and prognosis of intrahepatic CCA 4. An integrated genome-wide and whole transcriptome sequence study reported the ectopic activation of the FGF/FGFR2-mediated signaling pathway in CCA, which could serve as a potential therapeutic target 5. However, data on the regulation of FGF7 in CCA development are limited, to the best of our knowledge.

Long non-coding RNAs (lncRNAs), which are >200 nucleotides in length, are non-coding RNA molecules. Aberrant lncRNA expression has been observed in the pathological development of CCA 6. For example, in a previous study, a total of 2,392 downregulated and 2,773 upregulated lncRNAs were observed in intrahepatic CCA tissues, compared with normal tissues 7. Long intergenic non-protein coding RNA 630 (LINC00630), which is 2,117 bp in length and located at chromoesomeXq22.1, is mainly found in the cytoplasm, does not contain any transcripts and has been confirmed to have no protein-coding ability 8. LINC00630 has been demonstrated to be positively associated with tumor size, increase cell proliferation and promote non-small cell lung cancer cell migration 8. However, the mechanism through which LINC00630 mediates CCA development remains poorly understood, to the best of our knowledge. The aim of the present study was to investigate whether LINC00630 promoted CCA cell proliferation, migration and invasion by mediating the microRNA (miRNA/miR)-199a/FGF7 signaling axis.

## Materials and Methods

### Sample collection

The present study was approved by the Ethics Committee of The First Affiliated Hospital of Gannan Medical University (Ganzhou, China), and was conducted according to the Principles of the Declaration of Helsinki. All patients included in the present study provided written informed consent. A total of 36 pairs of CCA samples and their corresponding adjacent non-tumor tissues were obtained from patients who were diagnosed with CCA between April 2015 and October 2019. These patients did not receive any chemotherapy prior to being recruited for the study. The tissue samples were confirmed and histologically diagnosed as CCA by two pathologists. Tissue samples were prepared and frozen in liquid nitrogen, and stored at -80˚C until required for further use. The clinicopathological data of patients with CCA are shown in Table 1.

### Cell culture

HuCCT1, RBE, CCLP-1 and HCCC-9810 CCA cell lines, and human intrahepatic biliary epithelial cells (HIBECs) were obtained from The Cell Bank of Type Culture Collection of The Chinese Academy of Sciences. Cells were cultured in RPMI-1640 medium (Gibco; Thermo Fisher Scientific, Inc.) containing 10% FBS (Invitrogen; Thermo Fisher Scientific, Inc.) and 100 U/ml penicillin and streptomycin (Invitrogen; Thermo Fisher Scientific, Inc.) in a humidified incubator with 5% CO_2_ at 37˚C.

### Cell transfection

Three short hairpin RNAs [shRNAs/sh; sh-LINC00630-1 (5'-GAAGGAGTAGTTACGTAGA-3'), sh-LINC00630-2 (5'-GCTTATCAACATACTTCACTA-3') and sh-LINC00630-3 (5'-GGCTGTTACGTGAAGGAGAGA-3')] and a scrambled negative control (NC) shRNA (sh-NC; 5'-UGUGAGAUGCAGCCUCUAC-3') as the NC were purchased from Shanghai GeneChem Co., Ltd. These shRNAs were inserted into a pGPH1/Neo vector (Shanghai GenePharma Co., Ltd.). Next, the constructed pGPH1/Neo vector was prepared and transfected into CCA cells using Lipofectamine^®^ 3000 (Invitrogen; Thermo Fisher Scientific, Inc.), following the manufacturer's instructions. Cells were transfected using sh-LINC00630 or sh-NC for 48 h prior to further experiments. Neomycin (Sigma-Aldrich; Merck KGaA; cat. no. 1405-10-3; 400 μg/μl) was used to select the stably transfected cells for 4 weeks.

miR-199a mimics (5'-CCCAGUGUUCAGACUACGUGUUC-3' forward and 5'-ACAGGUAGUCUGAACAGUGGGUU-3' reverse), miR-199a inhibitors (5'-GAACAGGUAGACUGAACACUGGG-3') and miR-NC (5'-UUCUCCGAACGUCUCACGUTT-3' forward and 5'-ACGUGACACGUGCGGAGAATT-3' reverse) were obtained from Guangzhou RiboBio Co., Ltd. CCA cells, at 60% confluence (1x10^5^ cells/well), were transfected using Lipofectamine 3000, following the manufacturer's instructions. The concentrations of miR-199a mimics, miR-199a inhibitors and miR-NC were all 50 nM in the final transfection system.

Furthermore, pcDNA3.1-LINC00630 (Guangzhou RiboBio Co., Ltd.), pcDNA3.1-FGF7 vector (Guangzhou RiboBio Co., Ltd.) and the NC (empty vector) pcDNA3.1 vector were prepared and then transfected into CCA cells using Lipofectamine 3000, following the manufacturer's instructions. The transfected cells were cultured for 48 h in an incubator with 5% CO_2_ at 37˚C, prior to conducting further experiments.

### MTT assay

The transfected CCA cells (5x10^3^/well) were cultured in 96-well plates at 37˚C for 48 h. An MTT assay was conducted following the instructions of the MTT Cell Proliferation and Cytotoxicity Assay Kit's manufacturer (cat. no. C0009S; Beyotime Institute of Biotechnology). Briefly, MTT (0.5 mg/ml per well) was added and co-incubated with cells at 37˚C for 4 h. Next, the formazan crystals were dissolved in DMSO (150 μl; cat. no. D8418; Sigma-Aldrich; Merck KGaA) in the dark. Absorbance was detected at a wavelength of 490 nm using a microplate reader (Thermo Fisher Scientific, Inc.).

### Reverse transcription-quantitative PCR (RT-qPCR)

TRIzol^®^ reagent (Invitrogen; Thermo Fisher Scientific, Inc.) was used for total RNA extraction, following the manufacturer's instructions. Then, 2 μg RNA was used for the reverse transcription of RNA to cDNA using M-MLV reverse transcriptase (Promega Corporation). qPCR was conducted using Power SYBRs Green PCR Master Mix (Applied Biosystems; Thermo Fisher Scientific, Inc.) to detect the mRNA expression of LINC00630 and FGF7. miR-199a expression was detected using the TaqMan™ MicroRNA Reverse Transcription kit and TaqMan Universal Master Mix II kit (Applied Biosystems; Thermo Fisher Scientific, Inc.). GAPDH and U6 were used as the endogenous reference for mRNA and miRNA, respectively. All forward and reverse primers were obtained from Biomics. The primer sequences used were as follows: LINC00630, 5'-TACCGTTATTATTTCCC-3' forward and 5'-TCCTAAGTATTGACCCT-3' reverse; FGF7, 5'-AATGACATGAGTCCGGAGCAA-3' forward and 5'-CCATAGGAAGAAAATGGGCTG-3' reverse; GAPDH, 5'-AGGTGAAGGTCGGAGTCAACG-3' forward and 5'-AGGGGTCATTGATGGCAACA-3' reverse; miR-199a, 5'-CGCGCCCAGTGTTCAGACTAC-3' forward and 5'-AGTGCAGGGTCCGAGGTATT-3' reverse; U6, 5'-CTCGCTTCGGCAGCACA-3' forward and 5'-AACGCTTCACGAATTTGCGT-3' reverse. The expression of miRNA and mRNA was expressed as a fold change using the 2^-∆∆Cq^ method 9.

### Western blotting

The total proteins from tissues and cultured cells were extracted in ice-cold RIPA lysis buffer (Beyotime Institute of Biotechnology), and a BCA protein assay kit (Beyotime Institute of Biotechnology) was used to determine protein concentration. Total protein (25 μg) from each sample was subjected to 10% SDS-PAGE and then electrophoretically transferred onto PVDF membranes (MilliporeSigma). Membranes were blocked with TBS, which contained 5% non-fat milk for 1 h at room temperature. The membranes were then incubated with primary antibodies against vimentin (cat. no. 5741; dilution, 1:1,000; Cell Signaling Technology, Inc.), E-cadherin (cat. no. 3195; dilution, 1:1,000; Cell Signaling Technology, Inc.), FGF7 (cat. no. HPA043605; dilution, 1:1,000; Sigma-Aldrich; Merck KGaA) and GAPDH (cat. no. SAB1410512; dilution, 1:1,000; Sigma-Aldrich; Merck KGaA) at 4˚C overnight, followed by incubation with a secondary antibody conjugated with peroxidase (cat. no. AP510; dilution, 1:2,000; Sigma-Aldrich; Merck KGaA) for 1 h. Protein band values were detected using the enhanced chemiluminescence detection system (Bio-Rad Laboratories, Inc.) and Quantity One software v4.6.2 (Bio-Rad Laboratories, Inc.).

### Transwell migration and invasion assays

CCA cells were harvested after transfection for 24 h and prepared for cell suspension in a serum-free culture medium (3x10^4^ cells/ml). The cell suspensions were added to the upper chambers of the Transwell plates at room temperature, while RPMI-1640 medium supplemented with 20% FBS was added to the lower chambers. For the invasion assay, Matrigel (MilliporeSigma) was used to precoat the Transwell membranes overnight at room temperature. Following incubation at 37˚C for 24 h, the migratory and invasive cells were harvested, washed and stained with 0.5% crystal violet (Sigma-Aldrich; Merck KGaA) for 15 min. Finally, the cells in each sample were counted using an inverted light microscope (Olympus CK-40; Olympus Corporation) at a magnification of x400.

### Dual-luciferase reporter assays

The online prediction software StarBase v2.0 (http://starbase.sysu.edu.cn) and TargetScan 7.2 (http://www.targetscan.org) were used to identify the miRNA target of LINC00630 and target gene for miR-199a, respectively. The recombinant luciferase plasmids were constructed by cloning the sequences of wild-type (WT) LINC00630 and 3'-untranslated region (UTR) of FGF7 into the pGL-3 luciferase basic vector (Promega Corporation). In addition, their mutant (MUT) types with MUT binding sites for miR-199a were constructed (MUT-LINC00630 and MUT-FGF7). Each constructed plasmid was transfected into CCA cells with miR-199a mimics or miR-NC using Lipofectamine 3000. Following incubation for 48 h at 37˚C, the Glomax 96 luminometer (Promega Corporation) was used to detect the firefly and *Renilla* luciferase activities, according to the manufacturer's instructions. The firefly luciferase reporter activity was normalized to the *Renilla* luciferase activity.

### RNA immunoprecipitation (RIP) assay

An RIP assay was performed to explore the direct interaction between LINC00630 and miR-199a using an RIP kit (MilliporeSigma), following the manufacturer's instructions. CCA cells (2x10^7^ cells) were lysed using RIPA lysis buffer (Beyotime Institute of Biotechnology) and then incubated with magnetic beads precoated with antibodies against Argonaute 2 (Ago2; cat. no. MABE56; Sigma-Aldrich; Merck KGaA); anti-immunoglobulin G (IgG; cat. no. I5131; Sigma-Aldrich; Merck KGaA) was used as the negative control. TRIzol reagent was used for the RNA extraction and the RNA enrichment was determined using RT-qPCR. Finally, the expression levels of LINC00630 and miR-199a in the anti-IgG and anti-Ago2 groups were compared.

### Statistical analysis

All data are presented as the mean ± SD. SPSS 20.0 software (IBM Corp.) was used for the statistical analysis. Pearson's χ^2^ test or Fisher's exact test was used to analyze the clinicopathological data of patients. Differences between CCA and normal tissues were analyzed using a paired Student's t-test. One-way ANOVA followed by a Tukey's post hoc test was used to compare statistical differences among multiple groups. An unpaired Student's t-test was used to compare differences between two groups. Pearson's correlation coefficient was used to calculate the correlation. P<0.05 was considered to indicate a statistically significant difference.

## Results

### LINC00630 is upregulated in CCA tissues and cultured cells

**Figure 1:**
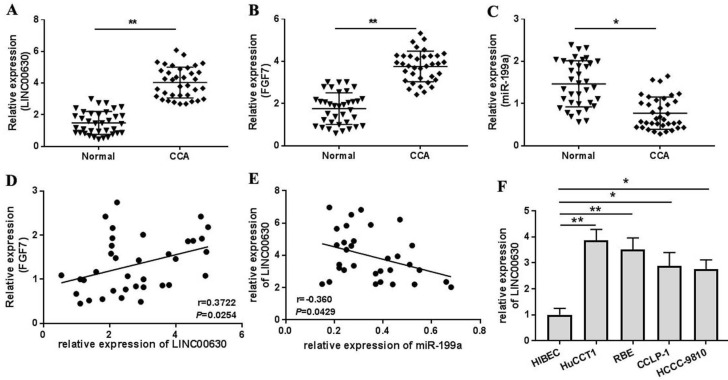
LINC00630 expression in CCA tissues and cultured cells. Expression of (A) LINC00630, (B) FGF7 and (C) miR-199a in CCA tissues (n=36) and adjacent normal tissues (n=36) was determined using RT-qPCR. Pearson's correlation analysis between LINC00630 and (D) FGF7 or (E) miR-199a expression was conducted in CCA tissues. (F) Expression of LINC00630 in human CCA cell lines (HuCCT1, RBE, CCLP-1 and HCCC-9810) and HIBECs was determined using RT-qPCR. All experiments were repeated three independent times, and data are presented as the mean ± SD. ^*^P<0.05 and ^**^P<0.01. CCA, cholangiocarcinoma; FGF7, fibroblast growth factor 7; miR, microRNA; RT-qPCR, reverse transcription-quantitative PCR; HIBECs, human intrahepatic biliary epithelial cells; LINC00630, long intergenic non-protein coding RNA 630.

Firstly, LINC00630, miR-199a and FGF7 expression in CCA tissues was detected using RT-qPCR. As shown by the results, LINC00630 (Fig. 1A) and FGF7 (Fig. 1B) expression was significantly increased and miR-199a expression (Fig. 1C) was decreased in CCA tissues, compared with those in the adjacent normal tissues. Pearson's correlation analysis identified a positive correlation between LINC00630 and FGF7 expression (Fig. 1D) and a negative correlation between LINC00630 and miR-199a expression (Fig. 1E). To determine the clinical significance of high expression of LINC00630 in CCA tissues, patients were stratified according to the median expression level of LINC00630 in CCA tissues. In addition, LINC00630 expression was significantly associated with histological differentiation, TNM stage and lymphatic invasion (Table 1). LINC00630 expression was significantly increased in all CCA cell lines (Fig. 1F), compared with that in HIBECs. Furthermore, HUCCT1 and RBE cells exhibited the highest levels of LINC00630 expression and were selected for the following experiments.

### LINC00630 knockdown suppresses cell proliferation, migration and invasion

**Figure 2:**
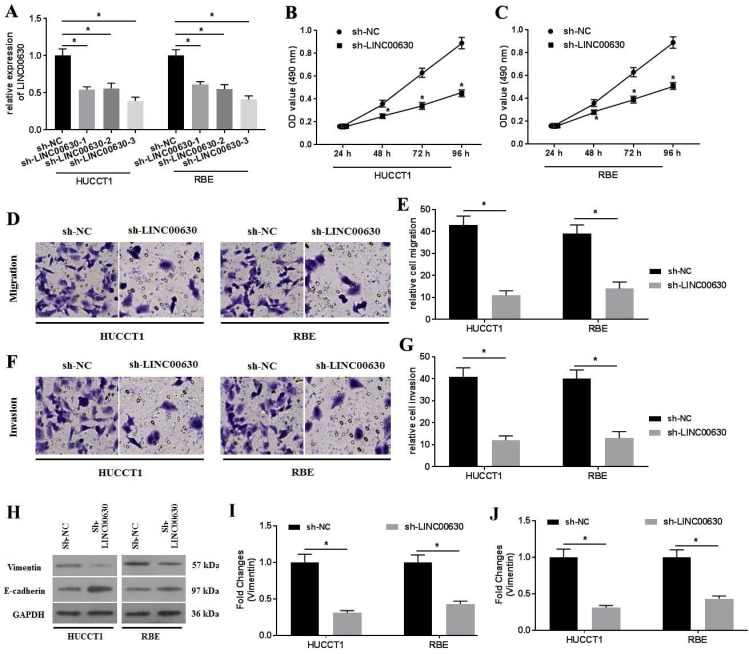
LINC00630 knockdown decreases HUCCT1 and RBE cell proliferation, migration and invasion. (A) Efficiency of sh-LINC00630 knockdown was determined using reverse transcription-quantitative PCR. An MTT assay was performed to determine (B) HUCCT1 and (C) RBE cell viability. A Transwell assay was performed to detect the (D and E) migration and (F and G) invasion of sh-LINC00630-transfected HUCCT1 and RBE cells. (H) Expression of vimentin and E-cadherin was determined using western blotting. (I and J) Intensity values of the protein bands were calculated. All experiments were repeated three independent times, and data are presented as the mean ± SD. ^*^P<0.05 and ^**^P<0.01. LINC00630, long intergenic non-protein coding RNA 630; sh-, short hairpin RNA.

To explore the roles of LINC00630 in CCA cells, three shRNAs (sh-LINC00630-1, sh-LINC00630-2 and sh-LINC00630-3) against LINC00630 were constructed and transfected into HUCCT1 and RBE cells. The transfection efficiency was detected using RT-qPCR (Fig. 2A). Transfection with all shRNAs significantly decreased LINC00630 expression in HUCCT1 and RBE cells. Sh-LINC00630-3 exhibited the highest activity, thus it was selected for the following experiments. LINC00630 knockdown decreased HUCCT1 (Fig. 2B) and RBE (Fig. 2C) cell proliferation at 48-96 h. The migration and invasion assays indicated that sh-LINC00630 transfection significantly decreased HUCCT1 and RBE cell migration (Fig. 2D and E) and invasion (Fig. 2F and G). In addition, LINC00630 knockdown decreased vimentin expression and increased E-cadherin expression (Fig. 2H-J). By contrast, LINC00630 overexpression through pcDNA3.1-LINC00630 transfection (Fig. S1A) significantly increased cell proliferation (Fig. S1B-C), migration (Fig. S1D-E) and invasion (Fig. S1F-G) and reversed the expression of vimentin and E-cadherin (Fig. S1H-J) in HUCCT1 and RBE cells.

### LINC00630 interacts with miR-199a

**Figure 3:**
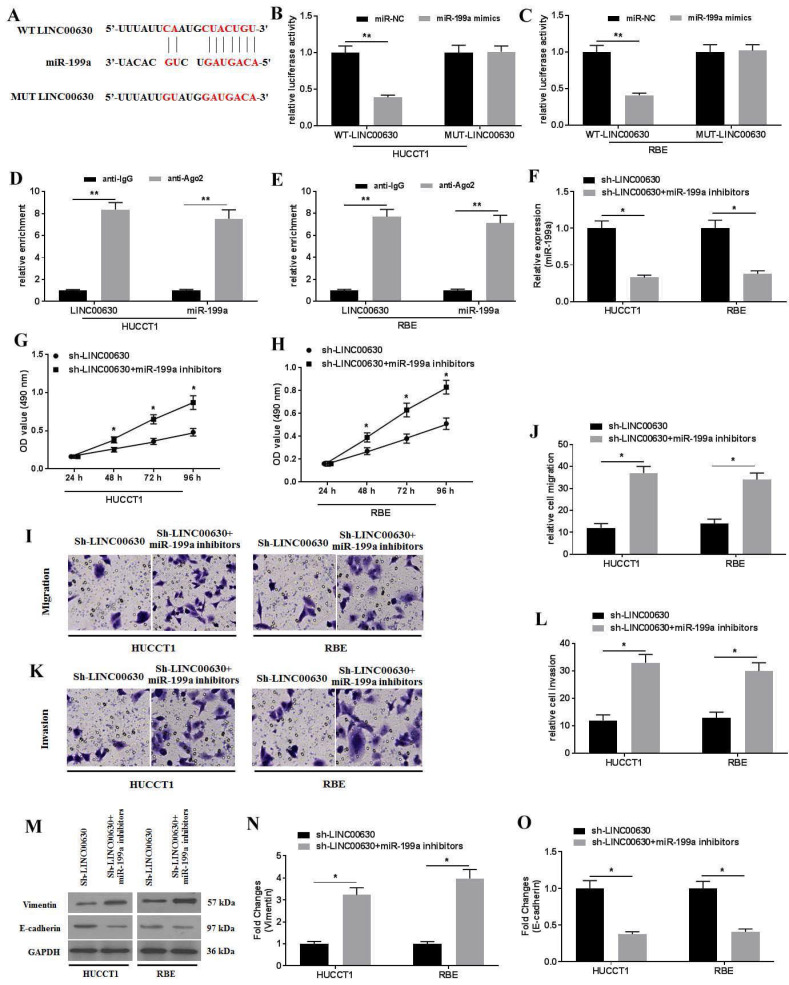
miR-199a is a target of LINC00630. (A) Potential interaction between LINC00630 and miR-199a was predicted using StarBase 2.0 database. The relative luciferase activity was detected in (B) HUCCT1 and (C) RBE cells co-transfected with both WT/MUT-LINC00630 and miR-199a mimics/miR-NC. The firefly luciferase reporter activity was normalized to *Renilla* luciferase activity. The interaction between LINC00630 and miR-199a was confirmed using a RNA immunoprecipitation assay in (D) HUCCT1 and (E) RBE cells. (F) Efficiency of miR-199a inhibitor transfection was determined using reverse transcription-quantitative PCR. An MTT assay was performed to determine cell viability in (G) HUCCT1 and (H) RBE cells. A Transwell assay was performed to detect the (I and J) migration and (K and L) invasion of sh-LINC00630- and miR-199a inhibitor-co-transfected HUCCT1 and RBE cells. (M) Expression of vimentin and E-cadherin was determined using western blotting. (N and O) Intensity values of the protein bands were calculated. All experiments were repeated three independent times, and data are presented as the mean ± SD. ^**^P<0.01. miR, microRNA; WT, wild-type; MUT, mutant; LINC00630, long intergenic non-protein coding RNA 630; sh-, short hairpin RNA; NC, negative control.

To further explore the biological activity of LINC00630 in CCA cells, the possible miRNAs that interacted with LINC00630 were predicted using StarBase 2.0. The results identified that miR-199a could be a potential target of LINC00630 (Fig. 3A). Furthermore, the dual-luciferase reporter assay showed that the luciferase activity in the reporter containing the WT-LINC00630 was decreased following miR-199a mimic co-transfection. By contrast, no significant differences were observed in the relative luciferase activities in the luciferase reporter plasmids containing the MUT-LINC00630 vector (Fig. 3B and C). The RIP assay demonstrated that LINC00630 could interact with miR-199a (Fig. 3D and E). miR-199a knockdown (Fig. 3F) could rescue the effects of LINC00630 on proliferation (Fig. 3G-H), migration (Fig. 3I-J) and invasion (Fig. 3K-L), and reverse the expression of vimentin and E-cadherin (Fig. 3M-O) in CCA cells.

### miR-199a overexpression ameliorates the proliferation, migration and invasion of CCA cells

**Figure 4:**
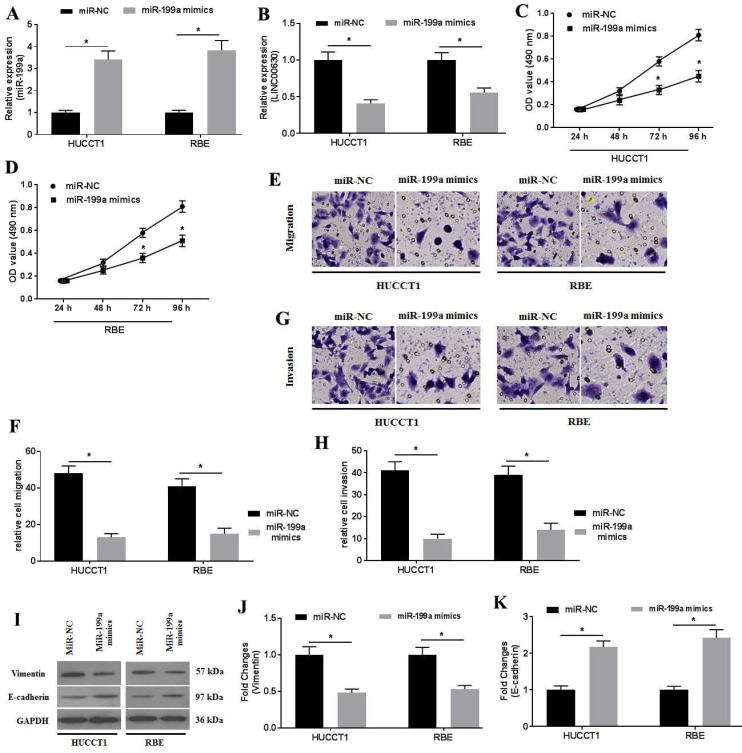
miR-199a overexpression ameliorates cholangiocarcinoma cell proliferation, migration and invasion. The expression level of (A) miR-199a and (B) LINC00630 in miR-199a mimic-transfected HUCCT1 and RBE cells was detected using reverse transcription-quantitative PCR. The proliferative activity of miR-199a mimic-transfected (C) HUCCT1 and (D) RBE cells was detected using a MTT assay. Transwell assays were performed to determine the (E and F) migration and (G and H) invasion of miR-199a mimic-transfected HUCCT1 and RBE cells. (I) Expression of vimentin and E-cadherin was determined using western blotting. (J and K) Intensity values of the protein bands were calculated. All experiments were repeated three independent times and data are presented as the mean ± SD. ^*^P<0.05. miR, microRNA; CCA, cholangiocarcinoma; LINC00630, long intergenic non-protein coding RNA 630.

To investigate the activity of miR-199a in CCA cells, miR-199a mimics were transfected into CCA cells. RT-qPCR was performed to determine miR-199a expression (Fig. 4A). miR-199a overexpression significantly suppressed LINC00630 expression (Fig. 4B). Furthermore, miR-199a-mimic transfection also decreased HUCCT1 and RBE cell proliferation (Fig. 4C and D), migration (Fig. 4E and F) and invasion (Fig. 4G and H). In addition, miR-199a-mimic decreased vimentin expression and increased E-cadherin expression (Fig. 4I-K) in HUCCT1 and RBE cells.

### FGF7 is a direct target of miR-199a

**Figure 5:**
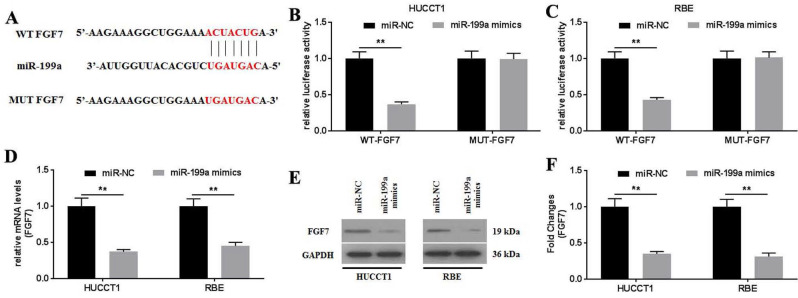
FGF7 is a direct target of miR-199a. (A) Potential interaction between miR-199a and FGF7 was predicted using TargetScan 7.2 database. The relative luciferase activity was detected in (B) HUCCT1 and (C) RBE cells co-transfected with both WT/MUT-FGF7 and miR-199a mimics/miR-NC. (D) mRNA expression of FGF7 was detected in miR-199a/miR-NC-transfected HUCCT1 and RBE cells. (E) Protein expression of FGF7 was detected in miR-199a/miR-NC-transfected HUCCT1 and RBE cells. (F) Fold changes of FGF7 protein expression. All experiments were repeated three independent times, and data are presented as the mean ± SD. ^**^P<0.01. FGF7, fibroblast growth factor 7; miR, microRNA; WT, wild-type; MUT, mutant; NC, negative control.

To further investigate how miR-199a affected the physiology of CCA cells, the targets of miR-199a were predicted using TargetScan v.7.2. The results showed that FGF7 might be a potential target of miR-199a (Fig. 5A). This was further confirmed using a dual-luciferase reporter assay (Fig. 5B and C). No statistical difference in the relative luciferase activity was observed between the luciferase reporter plasmids containing the MUT-FGF7. By contrast, the relative luciferase activities in the luciferase reporter plasmids containing the WT- FGF7 were significantly decreased. The mRNA and protein expression levels of FGF7 were determined and it was revealed that the miR-199a mimics significantly downregulated FGF7 mRNA (Fig. 5D) and protein (Fig. 5E and F) expression. Collectively, miR-199a may specifically target FGF7 to degrade it by binding to its 3'-UTR.

### FGF7 overexpression rescues the biological actions induced by LINC-00630 knockdown in CCA cells

**Figure 6:**
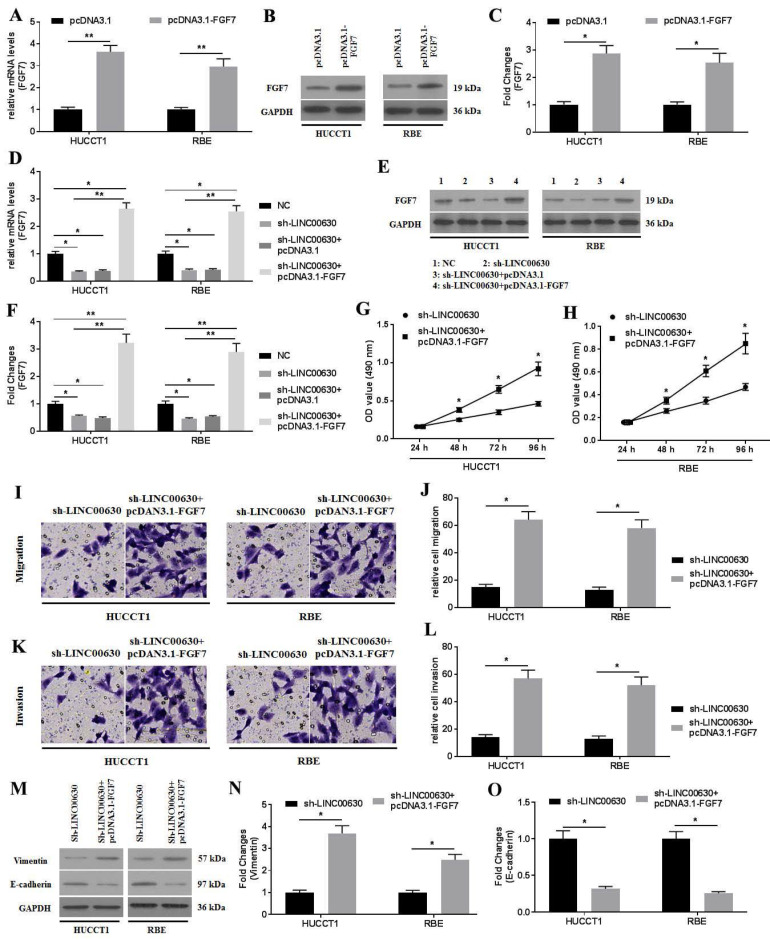
FGF7 overexpression improves the negative effects of LINC00630 knockdown on CCA cells. (A) mRNA expression of FGF7 was detected in HUCCT1 and RBE cells. (B and C) Protein expression of FGF7 was determined in HUCCT1 and RBE cells. mRNA (D) and protein (E and F) expression of FGF7 was detected in NC-, sh-LINC00630-, sh-LINC00630-pcDNA3.1- and sh-LINC00630-pcDNA3.1-FGF7-transfected HUCCT1 and RBE cells. Proliferation was detected in sh-LINC00630 and sh-LINC00630-pcDNA3.1-FGF7-transfected (G) HUCCT1 and (H) RBE cells using an MTT assay. (I and J) Migration and (K and L) invasion were determined in sh-LINC00630- and sh-LINC00630-pcDNA3.1-FGF7-transfected CCA cells. (M) Expression of vimentin and E-cadherin were determined using western blotting. (N and O) Intensity values of the protein bands were calculated. All experiments were repeated three independent times and data are presented as the mean ± SD. ^*^P<0.05 and ^**^P<0.01. FGF7, fibroblast growth factor 7; CCA, cholangiocarcinoma; LINC00630, long intergenic non-protein coding RNA 630; NC, negative control; sh-, short hairpin RNA.

First, FGF7 was overexpressed by transfecting pcDNA3.1-FGF7 into CCA cells, and RT-qPCR (Fig. 6A) and western blotting (Fig. 6B and C) were performed to confirm the success of the transfection, which was indicated by the increased expression of FGF7. Next, the co-transfection of sh-LINC00630-transfected CCA cells with pcDNA3.1-FGF7 was conducted. Similarly, the successful co-transfection was verified using RT-qPCR (Fig. 6D) and western blotting (Fig. 6E and F). FGF7 overexpression significantly enhanced the proliferation (Fig. 6G and H), migration (Fig. 6I and J) and invasion (Fig. 6K and L), and reversed the expression of vimentin and E-cadherin (Fig. 6M-O), rescuing these decreased biological actions induced by sh-LINC00630 transfection in CCA cells. Collectively, the overexpression of FGF7 could rescue the negative effects of LINC00630 knockdown in CCA cells.

## Discussion

CCA is a type of cancer with a low incidence rate, but a poor prognosis. Due to its unclear molecular mechanisms, current clinical diagnosis and treatment for CCA remain unsatisfactory. A large body of recent research has shown that lncRNAs play critical roles in the occurrence and development of various types of cancer 10, 11, including CCA 12. lncRNAs have been implicated in the splicing, export and translation of mRNAs and regulate the stability and post-translational modification of target proteins. In addition, lncRNAs can act as competing endogenous RNAs (ceRNAs) that interact with miRNAs and ameliorate their inhibitory effects on their target mRNAs 13. In the present study, the expression of LINC00630 was found to be significantly upregulated. LINC00630 knockdown could effectively decrease the proliferation, migration and invasion of CCA cells. Thus, it was suggested that the biological effects of LINC00630 in CCA cells might be associated with the regulation of the miR-199a/FGF7 axis.

There are ~20,000 protein-coding genes in the human genome, accounting for <2% of the entire genome. In total, >90% of the sequences are transcribed into RNAs, and most of these transcripts are non-coding RNAs 14. lncRNAs have been confirmed to play a critical role in the regulation of physiological processes in human diseases, particularly those involved in tumorigenesis and progression 15. However, the molecular mechanisms of lncRNAs in mediating the development of cancer are complicated. A recent study showed that five lncRNAs, (AL359715.5, AC006504.8, AC090114.2, AP00943.4 and hepatocellular carcinoma upregulated long non-coding RNA), have been screened for high-risk survival prediction in patients with CCA 16. lncRNA LINC00665 has been shown to be closely associated with poor prognosis and chemoresistance in patients with CCA. LINC00665 promotes sphere formation, migration, invasion and gemcitabine resistance 17. RHPN1 antisense RNA 1 functions to increase CCA cell proliferation and xenograft growth by regulating the miR-345/Yes1 associated transcriptional regulator axis 18. A study reports that LINC00630 may facilitate the binding of EZH2 to the promoter of BEX1 and the methylation of DNA, leading to epigenetic suppression in the radio-resistant CRC 19. Another study shows that LINC00630 increases the recruitment of E2F1 to the promoter of CDK2 and enhances CDK2 transcription, promoting cell proliferation and cell cycle progression and inhibiting cell apoptosis in HCC 20. Similarly, the present study showed that LINC00630 knockdown could ameliorate the proliferation, migration and invasion of CCA cells.

lncRNAs can exert their biological functions by sponging miRNAs, which degrade their target gene expression by binding to the 3'-UTR. It has been demonstrated that miR-18a can inhibit the activity of epithelial-to-mesenchymal transition by degrading suppressor of cytokine signaling 5 in CCA cells. However, miR-18a can be sponged by lncRNA cancer susceptibility 22 (CASC2), and the suppression of CASC2 expression accelerates cell proliferation and migration 21. miR-520f, a prognostic indicator, functions as a tumor suppressor in CCA and can be inhibited by small nucleolar RNA host gene 20 22. Another study showed that miR-204 acts as a target of HOX transcript antisense RNA and inhibits cell proliferation, promotes xenograft tumor growth, and increases cell apoptosis and autophagy by targeting high mobility group box protein 1 in CCA cells 23. In the present study, miR-199a was downregulated in CCA tissues and cultured cells. Pearson's correlation analysis found that miR-199a was negatively correlated with LINC00630. Notably, miR-199a overexpression could significantly ameliorate CCA cell proliferation, migration and invasion.

The FGF7 subfamily consists of FGF3, FGF7, FGF10 and FGF22, which are essential for organogenesis and tissue patterning and homeostasis 24. FGF family members mediate their physiological activities by interacting with FGFRs 25. The overexpression of FGFRs and their ligands has been observed in various types of human cancer. FGF7 knockdown has been shown to decrease retinoblastoma HXO-Rb44 cell invasion and proliferation, and increase their apoptosis 26. In addition, the overexpression of FGF7 enhances AKT activation and decreases reactive oxygen species generation, leading to increased U251 cell proliferation and decreased apoptosis. miR-144 targets and degrades FGF7, compromising the effects of FGF7, and promoting cell migration and invasion 27. In gastric cancer cells, AFAP1 antisense RNA 1 can promote proliferation, migration and invasion by enhancing FGF7 expression through sponging miR-155 28. In the present study, FGF7 was found to be significantly upregulated in CCA tissues and culture cells. FGF7 overexpression could effectively restore the reduction in proliferation, migration and invasion of CCA cells induced by LINC00630 knockdown.

## Conclusion

In conclusion, LINC00630 may be an oncogenic lncRNA in CCA, which enhances CCA progression by acting as a ceRNA that mediates FGF7 expression by sponging miR-199a. These findings suggest that LINC00630 may represent a potential therapeutic target for CCA.

## Supplementary Material

Supplementary figure.

## Figures and Tables

**Table 1:** The clinical pathological data of patients with CCA (n=36)

Variables	Total (36)	LINC00630 expression		*P*-value
		Low (17)	High (19)	
**Sex**				0.658†
Male	20	9	11	
Female	16	8	8	
**Age (years)**				0.731†
≤60	15	8	7	
>60	21	9	12	
**Histological differentiation**				0.042‡
Well or moderate	13	8	5	
Poor	23	9	14	
**TNM stage**				0.026†
I/II	17	9	8	
III/IV	19	8	11	
**Lymph node invasion**				0.049†
Negative	17	9	8	
Positive	19	8	11	
**Lymph node metastasis**				0.069‡
No	23	13	10	
Yes	13	4	9	

P‑values were calculated using Pearson's Chi‑squared test† or Fisher's exact test ‡.
