# Supplementary material for: LINC00630 promotes cholangiocarcinoma cell proliferation, migration and invasion by mediating the miR-199a/FGF7 axis
Source: J Cancer. 2022 Jan 4;13(3):975–86. doi: 10.7150/jca.66850 (PMC8824889; doi:10.7150/jca.66850)
Supplement: Supplementary file 1 — Supplementary figure. [file jcav13p0975s1.pdf]

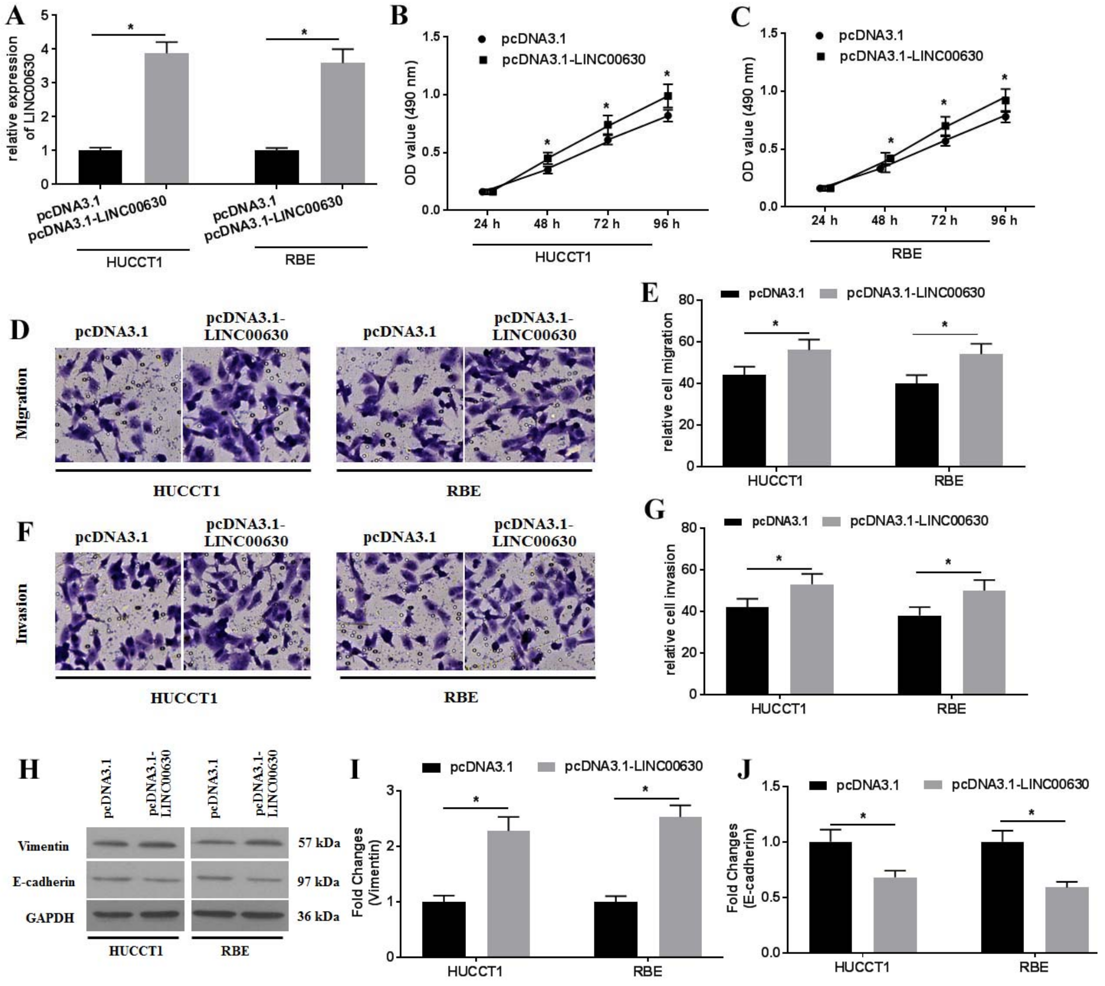

Figure 1S: The effects of LINC00630 overexpression on the proliferation, migration and invasion of HUCCT1 and RBE cells. (A) Efficiency of pcDNA3.1-LINC00630 transfection was determined using reverse transcription-quantitative PCR. An MTT assay was performed to determine (B) HUCCT1 and (C) RBE cell viability. A Transwell assay was performed to detect the (D and E) migration and (F and G) invasion of pcDNA3.1-LINC00630-transfected HUCCT1 and RBE cells. (H) Expression of vimentin and E-cadherin was determined using western blotting. (I and J) Intensity values of the protein bands were calculated. All experiments were repeated three independent times, and data are presented as the mean ± SD. *P<0.05 and **P<0.01.
